# Enabling animal rabies diagnostic in low-access areas: Sensitivity and specificity of a molecular diagnostic test from cerebral tissue dried on filter paper

**DOI:** 10.1371/journal.pntd.0008116

**Published:** 2020-03-06

**Authors:** Felana Suzah Rasolonjatovo, Hélène Guis, Malavika Rajeev, Laurent Dacheux, Lalaina Arivony Nomenjanahary, Girard Razafitrimo, Jean Théophile Rafisandrantantsoa, Catherine Cêtre-Sossah, Jean-Michel Heraud, Soa Fy Andriamandimby

**Affiliations:** 1 Rabies National Reference Laboratory, Virology Unit, Institut Pasteur de Madagascar, Antananarivo, Madagascar; 2 CIRAD, UMR ASTRE, 101 Antananarivo, Madagascar; 3 Faculty of Medicine, Department of Veterinary Medecine, Antananarivo, Madagascar; 4 Epidemiology and Clinical Research Unit, Institut Pasteur de Madagascar, Antananarivo, Madagascar; 5 ASTRE, Univ Montpellier, CIRAD, INRAE, Montpellier, France; 6 FOFIFA-DRZVP, Antananarivo, Madagascar; 7 Department of Ecology and Evolutionary Biology, Princeton University, Princeton, United States of America; 8 Institut Pasteur, Lyssavirus Epidemiology and Neuropathology Unit, National Reference Centre for Rabies, WHO Collaborating Center for Reference and Research on Rabies, Paris, France; 9 CIRAD, UMR ASTRE, F-97491 Sainte-Clotilde, La Réunion, France; US Department of Agriculture, UNITED STATES

## Abstract

Rabies is a lethal zoonotic encephalomyelitis that causes an estimated 59,000 human deaths yearly worldwide. Although developing countries of Asia and Africa bear the heaviest burden, surveillance and disease detection in these countries is often hampered by the absence of local laboratories able to diagnose rabies and/or the difficulties of sample shipment from low-access areas to national reference laboratories. Filter papers offer a convenient cost-effective alternative for the sampling, shipment, and storage of biological materials for the diagnosis of many pathogens including rabies virus, yet the properties of diagnostic tests using this support have not been evaluated thoroughly. Sensitivity and specificity of molecular diagnosis of rabies infection using a reverse transcription followed by a hemi-nested polymerase chain reaction (RT-hn-PCR) either directly on brain tissue or using brain tissue dried on filter paper were assessed on 113 suspected field animal samples in comparison to the direct fluorescent antibody test (FAT) recommended by the World Health Organization as one of the reference tests for rabies diagnosis. Impact of the duration of the storage was also evaluated. The sensitivity and the specificity of RT-hn-PCR i) on brain tissue were 96.6% (95% CI: [88.1–99.6]) and 92.7% (95% CI: [82.4–98.0]) respectively and ii) on brain tissue dried on filter paper 100% (95% CI: [93.8–100.0]) and 90.9% (95% CI: [80.0–97.0]) respectively. No loss of sensitivity of RT-hn-PCR on samples of brain tissue dried on filter paper left 7 days at ambient temperature was detected indicating that this method would enable analyzing impregnated filter papers sent to the national reference laboratory at ambient temperature within a 1-week shipment time. It could therefore be an effective alternative to facilitate storage and shipment of samples from low-access areas to enhance and expand rabies surveillance.

## Author summary

Dogs are responsible for 99% of human rabies deaths. Facilitating diagnostic of rabid dogs can help further identify i) people who were exposed to rabies in order to encourage them to seek post exposure prophylaxis and ii) other exposed animals to break the transmission chain (*i*.*e*. prevent them from further transmitting the virus to other people and animals). Yet, the reference diagnostic method for animal rabies requires brain samples to be collected post-mortem and shipped under temperature-controlled conditions. The shipping of such samples is complex in low-access areas, especially in low income countries, which are often those that bear the heaviest rabies burden. Filter papers offer a convenient alternative for biological sample shipment and storage. Here the efficiency of a molecular diagnostic method applied to brain tissue dried on filter paper is compared to one of the reference methods, the direct fluorescent antibody test. Our results show it has an excellent sensitivity (it does not miss any positive samples), even when filter papers are left 7 days at ambient temperature. These results let us foresee a cost-effective alternative facilitating shipment, storage and testing of samples from rabies suspected animals from low-access areas. This could considerably enhance and expand rabies surveillance in low-income countries, allowing a more comprehensive evaluation of rabies burden, and thus reinforcing arguments for allocating funds to rabies control policies.

## Introduction

Rabies is a lethal zoonotic encephalomyelitis caused by lyssaviruses affecting all mammals, including humans [1]. It is characterized by a highly variable incubation period and one of the highest fatality rates among infectious diseases. Indeed, once the symptoms appear, the outcome is always fatal for both animals and humans. Despite the entirely preventable nature of the disease, there are approximately 59 000 human deaths estimated annually worldwide [2].

In Madagascar, like in several African countries, rabies is endemic with 4 to 10 notified human cases and on average 54 animal cases per year between 2010 and 2015 confirmed by the National Reference Laboratory (NRL) [3]. The 31 anti-rabies medical centers provide post-exposure prophylaxis (PEP) free-of-charge for approximately 14,000 patients per year. These medical centers use the one-week 2 site intradermal injection regimen (also referred to as the updated or abridged modified Thai Red Cross protocol, or the Institut Pasteur du Cambodge regimen) (*i*.*e*. 2 intradermal injections of 0.1 mL at two sites (deltoids) on days 0, 3 and 7) [4]. Costs of dog vaccines are high for dog owners and coverage remains very low, even in Antananarivo city [5].

The NRL, the only laboratory performing rabies diagnosis in Madagascar, is located at Institut Pasteur of Madagascar (IPM), in Antananarivo city, Analamanga region. The routine diagnostic analysis of rabies suspected animal samples by the NRL showed that between 2010 and 2015, 383 (74.8%) of the 512 samples of known origin were from the Analamanga region. The remaining 129 samples came from 13 other regions, with on average of 1 sample sent per year per region. Eight of the 22 regions of Madagascar did not send any samples during that 6-year period. This suggests an important underestimation of the burden of rabies which in turn hinders resource attribution for prevention and control policies. Under-reporting of rabies is frequent, especially in low-income countries [6]. In Tanzania, for example, the incidence of human rabies is estimated to be more than 100-fold higher than the official human deaths reported [7]. In Madagascar, a projected burden of 246 to 758 human rabies deaths with current PEP averting 1307–4028 human deaths per year was recently estimated [8]. In animals, the underestimation of the burden could be even greater.

The absence or low number of samples from suspected animals originating from regions other than Analamanga sent for rabies investigation most probably arises from a combination of factors such as the lack of knowledge and awareness on the occurrence of the disease in the country, the low accessibility to health care centers, and difficulties in sample shipment. Rapid transport of samples, especially under a temperature-controlled supply chain to the laboratory can be particularly difficult in Africa because of the very low accessibility to remote rural areas and the limited access to appropriate transport containers. Most of the time, biological samples sent for the diagnosis of rabies to the NRL consist of large sections of brain tissue (or complete brain or head) transported in public buses, at ambient temperature.

Filter papers offer a convenient cost-effective alternative for the sampling, shipment and storage of biological materials such as blood, saliva or organ tissue for serological and molecular diagnosis of a wide range of viruses, bacteria and parasites [9–13]. Their small size makes them easy to pack and ship, and their conservation properties at ambient temperature facilitates shipping and storage. A limited number of studies have focused on the serologic [12, 14] or molecular diagnosis of rabies using filter papers [14–17]. The latter studies evaluated the feasibility and the methods for storing and extracting rabies virus RNA from filter papers. Yet, up to now, molecular diagnosis of rabies infection using samples stored on filter paper has not yet been thoroughly evaluated. In the present study, the level of sensitivity and specificity of molecular diagnosis of rabies infection using either directly brain tissue or using brain tissue dried on Whatman 903 filter paper were assessed on rabies-suspected field animal samples in comparison to the most widely used reference technique for rabies diagnosis, direct fluorescent antibody test (FAT), recommended by the World Health Organization (WHO) [18, 19]. The impact of the duration of storage was also evaluated. As a proof-of-concept, a limited number of samples were impregnated on filter paper in the field and sent to the NRL to be tested by PCR and their results were compared to FAT results on brain biopsies from the same animals carried out in parallel.

## Materials and methods

### Samples and diagnostic tests

As exact power calculations for dichotomous tests are complicated, the simplified three-step procedure described by Hess *et al*. [20] was used to compute sample size. To estimate a sensitivity of 99% with a power of 80% and a margin of error no greater than 0.05, at least 47 trues positive samples need to be included. To estimate a similar level of specificity with the same criteria, 47 true negative samples need to be included. Overall, 58 FAT positive (51 frozen and 7 fresh) and 55 FAT negative (49 frozen and 6 fresh) animal brain samples received at NRL were used in this study. The 113 (58+55) samples consisted in non-decomposed hippocampus samples received between 2014 and 2017 at the NRL from rabies suspected animals. Of the 113 samples, 91 originated from dogs, 18 from cats and 4 from cattle (see supplementary S1 Data for complete list of samples and S1 Fig and S2 Fig for STARD (Standards for Reporting Diagnostic Accuracy) flow diagrams).

**Fig 1:**
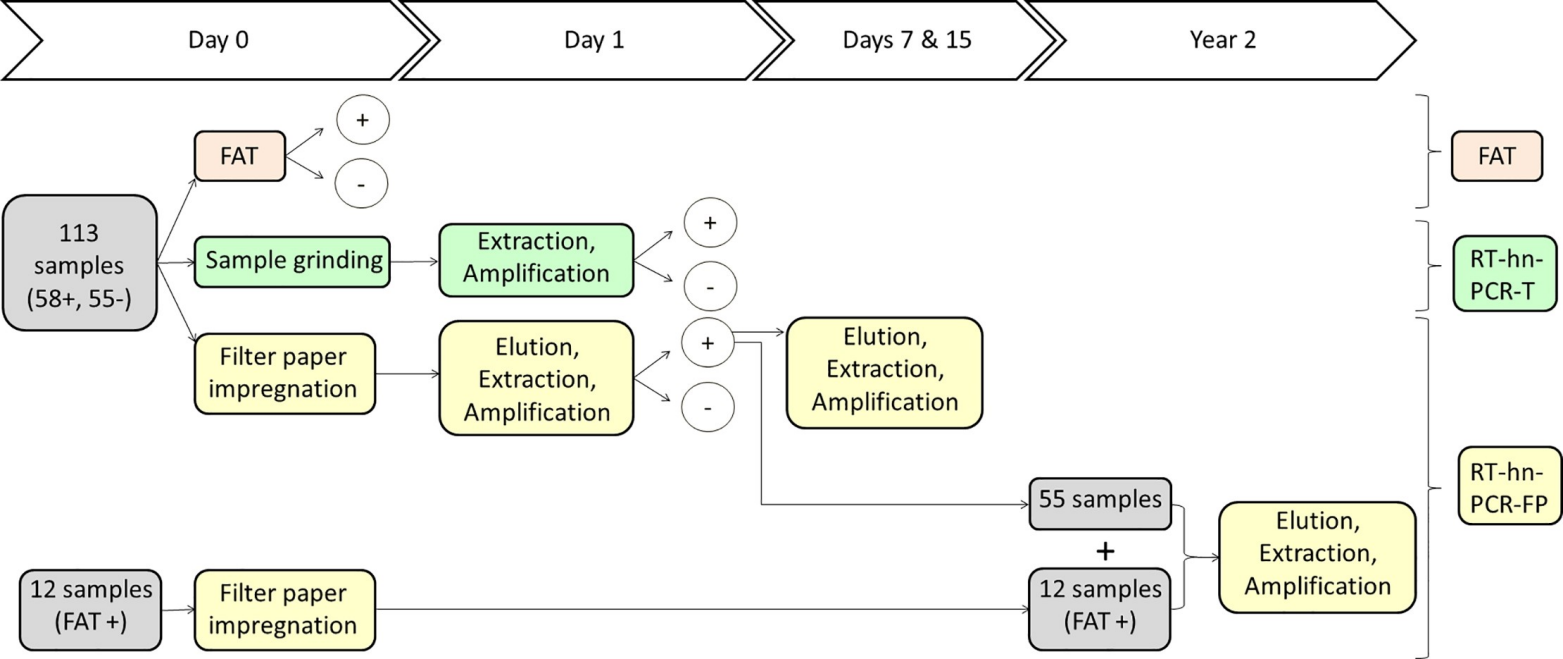
Diagnostic procedure. A total of 113 specimens were used during the study encompassing 58 FAT positive and 55 FAT negative samples. A total of 67 FAT positive samples were tested after 2 years of storage at ambient temperature.

Samples were processed in parallel for the three following diagnostic tests: i) direct FAT as reference in this study [18, 19] using anti-Rabies Nucleocapsid Conjugate (3572114, Bio-Rad, USA) ii) a reverse transcription followed by hemi-nested polymerase chain reaction on brain tissue [21] (here abbreviated as RT-hn-PCR-T) and iii) the same reverse transcription followed by hemi-nested polymerase chain reaction using brain tissue dried on filter paper (FP) (here abbreviated as RT-hn-PCR-FP) (Fig 1). On day 0 (D_0_), FAT was carried out and the samples were ground according to techniques described in [22] for RT-hn-PCR-T and impregnated on filter papers and left to dry 12 h for further RT-hn-PCR-FP on day 1. On day 1 (D_1_), RNA extractions and RT-hn-PCR were performed either directly on brain tissue or on FP, the latter requiring an initial (previous to RNA extraction) extra step of elution. On days 7 and 15 elution, RNA extraction and RT-hn-PCR were repeated for positive samples of brain tissue dried on FP to test the impact of storage duration. Fifty-five of these FAT positive samples and 12 other FAT positive samples (total 67) were used to impregnate filter papers which were stored at ambient temperature for further testing after 2 years (see supplementary S1 Data list).

### Brain tissue preparation for RT-hn-PCR-T

On D_0_, 100 mg of brain tissue (thawed or fresh) was homogenized in a Tissuelyser II (Qiagen, USA) in 900 μL of medium composed of 1% of L-glutamine (Reference W368401, Sigma-Aldrich, Germany), 1% of penicillin streptomycin (P4433-100Ml, Sigma-Aldrich, Germany), 1% of amphotericin B (Ref 15290026, GIBCO, USA), 40% of heat inactivated fetal calf serum (FCS) (Ref 12133C, Sigma-Aldrich, Germany) and 57% of minimum essential medium (MEM) (Ref 11095–080, Sigma-Aldrich, Germany) and further processed for RNA extraction.

### Sample preparation for RT-hn-PCR-FP: filter paper preparation

Using a single-use tongue depressor, a piece of 1 cm^3^ (650 ± 76.6 mg) of cerebral tissue was impregnated on a surface of 9 × 4 cm^2^ of Whatman 903 filter paper (Schleicher & Schuell, USA), as used by Wacharapluesadee *et al*. [16], corresponding to 18 mg on average of tissue per cm^2^. Filter paper was then dried for 12 h at room temperature (about 20° C) and enclosed in a paper envelope before being stored in a storage box at room temperature until use. On days 1, 7, and 15, two discs of 6 mm diameter (corresponding to 10 mg of brain tissue) were punched-out from the impregnated filter paper by using a 6 mm puncher and placed into a 2 ml microtube containing 200 μL of RNase free water (P1193, Promega, USA) for elution. Tubes were left for complete elution at room temperature for 30 minutes.

### RNA extraction and viral amplification

On D_1_, RNA was extracted either out of 100 μL of filter paper eluate or of ground brain tissue using TRIzol LS reagent (Life Technologies, USA) according to manufacturer’s recommendation. RNA was then subjected to reverse transcription (RT) and PCR amplification. Briefly, the pdN6 mix consisting of 2 μL of pdN6 random primers (200 mg/mL) (Ref C1181, Promega, USA) and 1 μL of RNase free water was added to 2 μL of extracted RNA and heated at 65°C for 10 min before being immediately placed on ice. The RT mix was made of 0.5 μL of RT-AMV (M5101, Promega, USA), 1 μL of dNTPmix (U1511, Promega, USA), 1 μL of RNasin (N2111, Promega, USA), 3 μL of RT-AMV buffer (M5101, Promega, USA) and 4.5 μL of RNase free water in a final volume of 15 μL. It was added to the pre-treated RNA and incubated for 90 min at 42°C to generate cDNA (complementary deoxyribonucleic acid).

The hemi-nested PCR was performed as previously described [21] with a minor modification. The initial protocol used a final volume of 50 μL and in this study, we used instead a final volume of 25 μL. Primers used in this protocol target conserved regions of the lyssavirus L-polymerase gene [23].

PCR amplified products were visualized on 1.5% agarose gels containing SYBR Safe (Ref S33102, Invitrogen, USA). The expected final sizes for the first and the second round of the hemi-nested RT-PCR were 320 bp and 250 bp respectively. Positive and negative control samples were included at each different step (extraction, reverse transcription, first and second PCR). The penultimate positive dilution of RNA extracted from a single known FAT-positive brain sample was used as a positive control throughout the study. Negative control of hemi-nested RT-hn-PCR consisted of known rabies negative brain tissue samples or known negative brain tissue impregnated on FP. For each step of RT and PCR, RNase free water was also used as a negative control.

### Interpretation of the results

FAT is the most widely used reference method for rabies diagnosis. FAT results were read by two staff members of the NRL. Samples were considered rabies positive by FAT when rabies virus nucleoprotein antigen (N) in brain smears was detected. They were considered rabies positive by RT-hn-PCR if amplicons with expected band size (250 bp) were obtained. In case of a discordant result between FAT and either RT-hn-PCR-T or RT-hn-PCR-FP, the sample was run a second time with FAT (FAT Repeat 1) and by two series of RT-hemi-nested PCR (repeat 1 (R1) and repeat 2 (R2)). For these repeated runs, samples underwent the whole procedure in duplicate for both RT-hn-PCR-T and RT-hn-PCR-FP (*i*.*e*. samples were ground (for RT-hn-PCR-T) or impregnated (for RT-hn-PCR-FP), extracted and amplified in duplicate). If one of the repeated tests still gave discordant results, mouse inoculation test (MIT) [24, 25] and the pan-rabies virus quantitative PCR (RT-qPCR) assay (TaqMan-based) adapted from Dacheux *et al*. [25], here called RT-qPCR TaqMan L, were used as confirmation tests. For the RT-qPCR TaqMan L, we used the same positive and negative controls as used for the hemi-nested PCR. In order to have a final confirmation for the discordant results, corresponding samples (brain suspension or RNA extraction deposited on FP or on Flinders Technology Associates filter paper cards—FTA cards) were sent to the National Reference Centre for Rabies (NRC-R) at Institut Pasteur, Paris, France, with positive and negative controls. Five techniques were used for this confirmation: two conventional RT-PCR with the original RT-hn-PCR L [21] and the RT-hn-PCR N [26], as well as three RT-qPCR with RT-qPCR TaqMan L [25], RT-qPCR SyBR L [25] and RT-qPCR SyBR N [19, 27]. Positive samples were confirmed after Sanger sequencing of the amplicons obtained with the original RT-hn-PCR L and/or with the RT-hn-PCR N, using the corresponding set of PCR primers [21, 26]. Partial nucleotide N (500 bp) and L (208 bp) gene sequences were aligned using ClustalX version 2.1 [28], combined to a representative dataset of sequences of other rabies viruses (RABV) belonging to the main phylogenetic clades [29], as well as a subset of RABV sequences from Madagascar. Multiple alignments were then used to construct the trees based on the maximum-likelihood approach with PhyML [30, 31] (see S3 Fig for the phylogenetic trees and for the complete description of the phylogenetic analysis).

Results of the first round of PCR (*i*.*e*. not the re-run results) were considered for calculating sensitivity and specificity of the two techniques. Sensitivity, specificity, positive and negative predictive values and 95% confidence intervals (CI) (exact Clopper-Pearson confidence intervals) were calculated (see supplementary S1 Table for STARD checklist).

### Impact of the FP storage on the molecular diagnosis of rabies virus

Fifty-eight samples that tested positive by RT-hn-PCR-FP on day 1 were tested for rabies on days 7 and 15. A set of 67 impregnated FAT positive samples (55 samples that tested positive by RT-hn-PCR-FP on day 1 and 12 other FAT positive samples) stored at ambient temperature were also tested at 24 months (Fig 1).

### First validation in the field

We also tested the use of FP for the first 10 samples collected in the field from Moramanga District (Alaotra Mangoro region) of Madagascar. These samples were collected using the straw method for brain tissue collection [32]. Paired brain biopsies were sampled from suspected rabid animals in order to perform in parallel FAT, RT-qPCR on FP, and a rapid immunochromatographic diagnostic test (RIDT) (Anigen, Bionote). The FP field collection method was different than the laboratory method due to ease of parallel sample collection for the different sample types. Briefly, approximately 10 mg of brainstem was mixed directly into the sample buffer provided in the Anigen kit as per the modified RIDT protocol recommended by Lechenne *et al*. [17]. After adding to the RIDT, 2–3 drops of the remaining solution were placed in each collection circle on the FP. The FP was allowed to dry for approximately 8–24 hours and then sent at ambient temperature to IPM, where the brain biopsies were used immediately for the FAT and the impregnated FP tested using the RT-qPCR TaqMan L [25]. As previously, we did not use the internal control described by Dacheux *et al*. (2016) but instead used the same positive and negative controls as used for the hemi-nested PCR.

## Results

### Sensitivity and specificity

Sensitivity and specificity of the RT-hn-PCR-T were 96.6% (95% CI: [88.1–99.6]) and 92.7% (95% CI: [82.4–98.0]) respectively (Tables 1 and 3). Sensitivity and specificity of RT-hn-PCR-FP were 100% (95% CI: [93.8–100.0]) and 90.9% (95% CI: [80.0–97.0]) respectively (Tables 2 and 3). Positive and negative predictive values must be interpreted with caution as they depend on prevalence. Here the proportion of positive samples used (58/113, 51.3%) is slightly inferior to the prevalence of rabies in samples shipped to the NRL over the past years (on average 62% between 2010 and 2015, with a minimum of 46.6% and maximum of 77.1% [33]).

**Table 1:** RT-hn-PCR-T results compared to FAT.

		FAT (Reference)		
		Positive	Negative	Total
RT-hn-PCR-T	Positive	56	4	60
	Negative	2	51	53
	Total	58	55	113

**Table 2:** RT-hn-PCR-FP results compared to FAT.

		FAT (Reference)		
		Positive	Negative	Total
RT-hn-PCR-FP	Positive	58	5	63
	Negative	0	50	50
	Total	58	55	113

**Table 3:** Evaluation of RT-hn-PCR-T and RT-hn-PCR-FP compared to FAT.

	RT-hn-PCR-T	RT-hn-PCR-FP
Sensitivity	96.6%	100%
[95% CI]	[88.1–99.6]	[93.8–100.0]
Specificity	92.7%	90.9%
[95% CI]	[82.4–98.0]	[80.0–97.0]
Positive Predictive Value	93.3%	92.1%
[95% CI]	[83.8–98.2]	[82.4–97.4]
Negative Predictive Value	96.2%	100%
[95% CI]	[87.0–99.5]	[92.9–100.0]
Positive likelihood ratio	13	11
Negative likelihood ratio	0.037	0
Accuracy	94.7%	95.6%

Eight samples had discordant RT-hn-PCR-T and/or RT-hn-PCR-FP results compared to FAT and were subsequently tested by duplicating FAT, RT-hn-PCR-T and RT-hn-PCR-FP tests and, if discordant results persisted, by confirming them with MIT and RT-qPCR TaqMan L (Table 4). Seven of these samples originated from dogs and one from a cat. Among these 8 samples, all FAT duplicated results (FAT R1) were identical to prior test and to FAT first testing results. Duplicated results of five samples (3693–15, 1751–16, 2557–14, 4255–16 and 2686–14) were concordant with FAT results. Duplicated results of three samples (3723–16, 2555–15 and 3656–15) remained discordant and were further tested by MIT, three RT-qPCR with RT-qPCR TaqMan L, RT-qPCR SyBR L, RT-qPCR SyBR N, as well as the two conventional RT-PCR with the original RT-hn-PCR L and the RT-hn-PCR N (Table 4). Duplicated RT-hn-PCR-T results of sample 3723–16 remained negative, but all other results were positive and the virus strain identified as Malagasy RABV after sequencing and phylogenetic analysis. This sample was thus considered as a rabies positive sample. Sample 2555–15 was negative by FAT, MIT, RT-hn-PCR N, RT-qPCR SyBR L and RT-qPCR TaqMan L performed at NRC-R in France, but positive by RT-hn-PCR-T, RT-hn-PCR-FP, RT-qPCR TaqMan L performed at NRL in Madagascar and by RT-hn-PCR L and RT-qPCR SyBR N performed at NRC-R. Sequencing results also confirmed the detection of a Malagasy RABV strain. Finally, 3656–15 was initially positive by RT-hn-PCR-FP D1 and RT-qPCR TaqMan L on brain tissue but negative by FAT, RT-hn-PCR-T and MIT at NRL in Madagascar. This sample was further confirmed as negative for all 5 techniques undertaken at the NRC-R in France.

**Table 4:** Complete results for samples with discordant results between RT-hn-PCR and FAT.

Samples		Id		3693–15	3723–16	2555–15	1751–16	2557–14	4255–16	3656–15	2686–14
		Animal		dog	dog	dog	dog	dog	dog	cat	dog
		Type		frozen	fresh	frozen	frozen	frozen	fresh	frozen	frozen
NRL, Mada-gascar	Prior FAT test			+	+	-	-	-	-	-	-
	First testing	FAT		+	+	-	-	-	-	-	-
		RT-hn-PCR-T		-	-	+	+	+	+	-	-
		RT-hn-PCR-FP	D 1	+	+	+	+	+	-	+	+
			D 7	+	+	NT	NT	NT	NT	NT	NT
			D 15	+	+	NT	NT	NT	NT	NT	NT
	Dupli-cates	FAT	R1	+	+	-	-	-	-	-	-
		RT-hn-PCR-T	R1	+	-	+	-	-	-	-	-
			R2	+	-	+	-	-	-	-	-
		RT-hn-PCR-FP	R1	+	+	+	-	-	-	+	-
			R2	+	+	+	-	-	-	+	-
	MIT			NT	+	-	NT	NT	NT	-	NT
	RT-q PCR TaqMan L		T	NT	+	+	NT	NT	NT	+	NT
			FP	NT	NT	+	NT	NT	NT	NT	NT
NRC-R, France	RT-hn-PCR N			NT	+	-	NT	NT	NT	-	NT
	RT-hn-PCR L			NT	+	+	NT	NT	NT	-	NT
	RT-qPCR SyBR N			NT	+	+ (weak)	NT	NT	NT	-	NT
	RT-qPCR SyBR L			NT	+	-	NT	NT	NT	-	NT
	RT-qPCR TaqMan L			NT	+	-	NT	NT	NT	-	NT
	Sequencing			NT	+	+	NT	NT	NT	-	NT
**Conclusion**				**+**	**+**	**+**	**-**	**-**	**-**	inc.	**-**

Id: sample identification number; prior test: prior to this study; FAT: direct fluorescent antibody test; RT-hn-PCR: reverse transcription followed by hemi-nested PCR; RT-qPCR TaqMan: quantitative PCR using TaqMan technology; RT-qPCR SyBR N: quantitative PCR using SyBR green technology; T: using brain tissue; FP: using brain tissue dried on filter paper; L: targeting the L gene; N: targeting the N gene; D: day; R1: repeat 1; R2: repeat 2; Grey shade: discordant results with FAT; NT: not tested; inc.: inconclusive.

### Impact of the FP storage on the molecular diagnosis of rabies virus

RT-hn-PCR-FP enabled to detect rabies virus on the 58 true-positive filter papers stored at room temperature with the same level of 100% of sensitivity at day 7. At day 15, two samples out of 58 tested negative leading to 96.6% of sensitivity. At 24 months, all 67 samples tested were positive by RT-hn-PCR-FP.

### First field application of the use FP in the molecular diagnosis of rabies virus

The ten samples collected in the field in Moramanga were all positive by FAT, RT-qPCR TaqMan L and RIDT (see supplementary S2 Data for complete list of field samples from Moramanga), showing that the process of i) impregnation of FP in the field, ii) shipment to the NRL and iii) molecular testing in the laboratory was feasible.

## Discussion

The successful use of molecular diagnostic methods using brain tissue directly or brain tissue dried on filter paper or FTA cards, that are filter papers impregnated with patented chemicals designed to preserve nucleic acids, to detect different species of lyssavirus (including rabies virus) has been demonstrated by several studies [14–17, 34, 35]. Yet, to our knowledge, the sensitivity and specificity of these methods using field collected samples had not yet been thoroughly evaluated. Lechenne *et al*. [17] compared results from RT-qPCR on impregnated FTA cards to FAT but the limited sample size (39 samples that gave interpretable results for both techniques) lead to very large confidence intervals (*i*.*e*. a sensitivity of 94.3% (95% CI: [80.8–99.3]) and a specificity of 100% (95% CI: [39.8–100])).

Our study demonstrates that the sensitivity of our hemi-nested reverse transcriptase PCR on filter paper is extremely high (100%; 95% CI: 93.8–100.0) and that its specificity is also high (90.9%; 95% CI: 80.0–97.0). Compared to the sample size initially calculated, the number of true positive samples was sufficient to evaluate sensitivity with 80% power margin of error no greater than 0.05. Conversely, to reach the same power and to keep the same margin of error for specificity, 164 true negative samples should have been included.

It is noteworthy that these excellent sensitivity results were obtained using a majority (88.5%) of frozen samples given that the freezing-thawing step could have contributed to the degradation of the virus inducing a decrease of sensitivity. Furthermore, RT-hn-PCR could only be carried out on D_1_ for logistic reasons (access to laboratory equipment and sample processing time (including drying time for FP)), and this delay could also have negatively impacted sensitivity. Moreover, these results were obtained in artificial conditions of important laboratory work load involving multiple series of PCRs carried out on an important number of samples, which can increase the risk of human error. In real-life conditions at the NRL of Madagascar, because samples of rabies-suspected animals are shipped with a low frequency, samples are processed one by one. Sometimes it is more difficult to recover RNA from brain tissue dried on filter paper than directly from brain tissue because there can be a decrease of the quantity of extracted RNA when using filter paper for molecular diagnosis: a loss of 1 log was shown for Newcastle disease virus [36] and of 0.825 log for Chikungunya virus [9]. Conversely, Picard-Meyer *et al*. did not show a loss in RNA when using FTA for rabies virus [14]. Although our study did not assess a potential loss in RNA, the excellent sensitivity result suggests that this loss was either negligible or non-existent. This study also enabled to evaluate the sensitivity and specificity of RT-hn-PCR on brain tissue (RT-hn-PCR-T) and showed that both sensitivity (96.6% (88.1–99.6)) and specificity (92.7% (82.4–98.0)) were high. Sensitivity of RT-hn-PCR-T was slightly lower than that of RT-hn-PCR-FP, but the difference was not statistically significant. RT-hn-PCR was chosen for its high sensitivity and its availability at the NRL. Nested PCR was shown to enable viral RNA detection from dried brain tissue on filter paper stored for up to 222 days contrary to non-nested PCRs which were only able to detect viral RNA from filter papers stored for 28 days [16].

Overall eight samples had discordant results. The results on duplicates for five of these samples (3693–15, 1751–16, 2557–14, 4255–16 and 2686–14) were concordant suggesting that human errors such as contamination or confusion between samples could have caused these discordant results. Indeed, for the four FAT negative samples (1751–16, 2557–14, 4255–16 and 2686–14), all duplicated tests were negative, suggesting that the discordant results could be due to a contamination during the first tests. For sample 3693–15, only one test was discordant, again suggesting a human error during this test. Furthermore, risk of human errors could have been amplified by heavy workload. Indeed, during this study, dozens of samples were processed simultaneously. For routine surveillance or when processing filter papers impregnated in the field, samples were processed consecutively. Confusion between samples rarely occurs when good laboratory practices are respected. As for any other technique, the application of the good laboratory practices and training on biosafety would be imperative if this technique were to be implemented for routine surveillance.

For three samples (3723–16, 2555–15 and 3656–15), duplicated results remained discordant. Sample 3723–16 was positive by FAT (prior test, first testing and duplicate), RT-hn-PCR-FP (D1, D7, D15, duplicates R1 and R2), MIT and RT-qPCR TaqMan L T but negative by RT-hn-PCR-T (first testing, duplicates R1 and R2). All 5 PCR carried out at NRC-R were positive as well as Sanger sequencing genotyping (Malagasy RABV), confirming that this sample was positive. Although we cannot rule out random errors, results of this rabies positive sample could suggest that RT-hn-PCR-T could be less sensitive for this sample than the other tests carried out or that PCR inhibitors or an inhibition due to overloading of nucleic acid after brain extraction occurred during the RT-hn-PCR-T (but not during RT-hn-PCR-FP nor during PCRs carried out at NRC-R). Sample 2555–15 was negative by FAT (prior test, first testing and duplicate) and MIT but positive by hemi-nested PCR tests (RT-hn-PCR-T, RT-hn-PCR-FP D1, RT-hn-PCR-T duplicates R1 and R2, RT-hn-PCR-T duplicates R1 and R2) and by RT-qPCR TaqMan L (FP and T). RT-qPCR TaqMan L results showed that the viral load was low (ct = 32 when using brain tissue and ct = 37.6 when using FP). Results obtained at NRC-R were also discordant: RT-hn-PCR N and RT-qPCR SyBR L and RT-qPCR TaqMan L were negative whereas RT-hn-PCR L was positive and RT-qPCR SyBR N was weakly positive. Sanger sequencing of the amplicons at NRC-R confirmed the Malagasy origin of the RABV. Based on these results, we can hypothesize that this sample was positive but with a low viral load. The latter could explain the discrepancies obtained with different techniques, reflecting their difference of sensitivity for this sample. According to his owner, this dog didn’t have any clinical symptoms when it was euthanized after having bitten someone. This information is compatible with the hypothesis that the animal was at an early stage of the infection, thus presenting a low viral load, which could have increased over time if it had not been euthanized. Sample 3656–15 was negative by FAT (prior test, first testing and duplicate), RT-hn-PCR-T (first testing, duplicates R1 and R2) and MIT but positive by RT-hn-PCR-FP (D1, duplicates R1 and R2) and RT-qPCR TaqMan L FP when tested at NRL laboratory (ct value of 35). However, all other tests performed at the NRC-R were negative. This sample originated from a cat which was described by the veterinarian as having symptoms suggestive of a paralytic form of rabies. This sample could have originated from a rabid animal with a very low viral load that was not detected by FAT and MIT. FAT and MIT are highly sensitive and specific but they are not perfect. Sensitivity and specificity of FAT are considered to be between 96% and 99% [37]. FAT sensitivity can vary depending on the expertise of the examiner, quality of the conjugate, equipment, appropriate tissue sampling and sample conservation [19, 32]. Several freeze-thaw cycles could also have further hindered analyses carried out at NRC-R on a sample with an initial very low viral load. Another hypothesis may be that during manipulation contamination occurred, yet of the fact that four tests yielded positive results is not in favor of this hypothesis. Results on this sample finally remained inconclusive.

Although it was not the main objective, this study showed that viral RNA could be detected from filter papers stored for up to 2 years at room temperature. Sensitivity of the method was 100% on day 1 and day 7 but decreased to 96.7% on day 15 where 2 samples (out of 58 positive) turned negative. These 2 latter samples were frozen samples. It is noteworthy that one of these two samples already had weak signals when tested by FAT on day 0 and by RT-hn-PCR-FP on day 1. Conversely, after two years, all 67 samples stored at ambient temperature tested positive. Two studies have demonstrated the possibility of rabies RNA detection over longer storage periods (43 and 222 days) [14, 16]. Our slightly inconsistent results on the effect of storage of filter papers could be due to random errors, or for one sample, to an initial very low viral load. Results would therefore need to be confirmed by other studies. Thus, from a public health point of view, we can conservatively affirm that sending impregnated filter papers to the NRL within a one-week delay would not impact the sensitivity of the RT-hn-PCR-FP. Longer storage periods are probably possible but more thorough investigations should be carried out. In particular, before field implementation of FP as a support for molecular testing (in the lab) of rabies virus, we recommend to further assess the combined effect of time and temperatures (*i*.*e*. testing storage at higher temperatures). The use of RT-qPCR on FP could also be compared to RT-hn-PCR-FP as RT-qPCR is expected to be more sensitive and less prone to contamination.

Preliminary results relative to samples from Moramanga showed it was possible to successfully i) carry out FP impregnation in the field, ii) ship impregnated FP to a central laboratory and iii) carry out molecular testing of these samples in the laboratory. Although Moramanga city in Moramanga district is not very far from Antananarivo city (approximately 110 km), villages in the district are poorly connected through dirt roads and paths and often involve walking to reach serviceable roads. These field results should be strengthened by collecting a greater number of samples and by asking different teams in multiple locations to test the process. The main practical drawback from this field experience was that the quantity of brain obtained using the straw method was small, limiting further testing of the samples. More brain samples should be collected on each animal to overcome this limit.

Overall, the excellent sensitivity of RT-hn-PCR-FP demonstrated in our study shows the high potential for the use of filter paper in rabies surveillance, especially in low-income countries with poor road infrastructures, where shipping rabies suspected brain tissue specimens can be difficult. Excellent sensitivity is a compulsory requirement when test results of rabies suspected animals impact the post-exposure prophylaxis (PEP) continuation in humans since false negative results may lead to PEP interruption and death of the exposed person. Yet, given that molecular diagnostic capacities in Madagascar are extremely limited (the NRL is the only laboratory performing rabies molecular diagnosis in the country), and given the short (one-week) [4] and free-of-charge PEP protocol in place in Madagascar, the main objective of using impregnated FP in Madagascar is not to guide PEP but rather to facilitate sample collection from low-access areas which have never or seldom submitted samples of rabies suspected animals. The fact that sensitivity is not impacted by a 7-day storage period at room temperature lets us foresee a possible coverage of most if not all the island.

Although we have demonstrated the feasibility to impregnate filter papers with brain tissue in the field and to ship them to the laboratory for subsequent molecular diagnosis, several steps are necessary before considering expanding this method to the entire country. The first step to tackle concerns methods to access brain tissue of rabies suspected animals. In Madagascar, this is most frequently done by breaking the animal’s skull. Yet, because this method is hazardous, especially in the field, alternative tissue collection methods consisting in using a straw via the trans-orbital or trans-occipital route are recommended. Vaccination and training of the public health officers and field staff on these methods would also need to be implemented in Madagascar.

Because Whatman type FP do not guarantee pathogen inactivation, the presence and infectiousness of infectious particles in brain tissue impregnated filter papers warrants further assessment. Indeed, 5 filter papers impregnated with infected brain tissue were tested by cell culture isolation (using Neuro-2A ATCC CCL-131 cell lines) 17 hours after drying and 3 of the 5 samples were still infectious. A more thorough study with a statistically supportive sample size should be set up to confirm these results and to assess the effect of storage duration on infectiousness of brain tissue impregnated on FP. Conversely, studies using FTA cards have shown that rabies virus [14], like other viruses such as avian influenza Newcastle disease viruses [13, 36], were completely inactivated within a few hours after adsorption to FTA cards at room temperature. A similar study to evaluate the sensitivity and specificity of molecular diagnosis using brain impregnated FTA cards instead of Whatman filter papers, as done with RT-qPCR [17], could be carried out using a statistically supportive sample size, although costs would increase slightly. In any case, a parcel with a triple packaging (consisting of one sealed envelope into one plastic bag, itself into another sealed envelope for example) is necessary to ensure safe shipment [38].

The excellent sensitivity and very high specificity of rabies RT-hn-PCR-FP compared to the FAT could be a cost-effective alternative for facilitating sample shipment and storage of rabies suspected animals from low-access areas to the NRL. This would greatly help enhance and expand surveillance coverage, in turn improving accuracy of reported data. Furthermore, FP and FTA cards also have the noteworthy advantage of enabling molecular characterization of rabies virus isolates [14] and establishing phylogenetic relationships [39] which help improve our understanding of rabies circulation across larger spatial scales and also at micro-scales[40] as they can help determine if the virus persists or is re-introduced into areas under study. This is particularly important in times of dog-transmitted human rabies elimination programs such as the “Zero by thirty” (zero human deaths by 2030) objective launched by WHO, FAO (Food and Agriculture Organization of the United Nations), OIE (World Organization for Animal Health) and GARC (Global Alliance for Rabies Control). Indeed, mass vaccination campaigns of dogs have to take into account risks of possible reintroduction of the virus from areas not yet covered by vaccination campaigns, and phylogenetic studies can help assess this risk as shown by Bourhy *et al*. [40], together with FP helping extend availability of samples from remote areas. Using FP or FTA cards can facilitate launching surveillance activities even in difficult contexts as shown in Liberia where, because of the absence of trained personnel, laboratory facilities and cold storage facilities in Liberia at the time, active rabies surveillance relying on the use of FTA cards to collect and preserve samples for subsequent molecular analysis was set up [41]. Unfortunately, activities were suspended shortly after the 2014 Ebola virus outbreak. Several steps including the training and vaccination of staff involved in sample collection and the logistics of supplying FP, personal protective equipment and appropriate packaging for shipment should be organized before considering implementation of FP in the field at a larger scale for rabies surveillance.

However, the implementation of RT-hn-PCR-FP for surveillance activities will increase the lab costs (because RT-hn-PCR are more expensive than FAT tests). Another argument against this technique might be that if NRL only receive brain tissue impregnated FP instead of brain tissue samples, rabies viral strains will no longer be available to sustain virus repositories. Yet NRL need to have access to virus repositories for immunological, virology, vaccine and epidemiological studies. A compromise might be that i) in areas of low-access, from which there are currently no samples being shipped, the use of FP as sample support is implemented and ii) when samples are collected from more accessible areas, brain biopsies are collected ideally alongside to (or if not possible, instead of) impregnated FP.

Other techniques, such as direct rapid immunohistochemistry test (dRIT) and RIDT, have been developed over the past decades to improve rabies diagnostic and surveillance. When brain tissue is available for testing, dRIT is an interesting alternative to FAT as, contrary to FAT, it does not require the acquisition of an expensive fluorescence microscope, and can thus facilitate the implementation of decentralized laboratories if appropriate supply and storage of reagents can be ensured [19]. Alongside FAT and PCR assays, dRIT is a recommended post-mortem diagnostic test for animals [37]. In countries with poor healthcare and transport infrastructures brain samples can be stored in a glycerol and phosphate buffer saline (PBS) solution to improve sample preservation. Yet, because of possible limited conservation under high temperatures (above 30° C), it is recommended, when possible, to keep samples refrigerated [37].

Apart from using FP (or FTA cards), another way to overcome problems due to sample storage and shipment in low-access areas is to use point of sample tests. RIDT such as Anigen have the strong advantage of being easy-to-use point of sample tests. However, they still need to be fully validated prior to use. They have good although not perfect sensitivity under laboratory conditions (95.3%, 95% CI: [84.2–99.4]) when using the modified protocol (without the dilution step in PBS) [17]. Although these results are promising, they cannot substitute current reference tests [17]. RIDT cassettes can be used for further confirmation by molecular testing, but their sensitivity was shown to be lower than when using FTA cards as support [17].

Given its excellent sensitivity and very high specificity, the use of FP as a support for the molecular diagnostic of rabies infection seems to be an effective alternative to facilitate storage and shipment of samples from low-access areas to enhance and expand surveillance. The improvement of rabies surveillance, particularly in low-income countries, allows foreseeing a more comprehensive evaluation of rabies burden, which would in turn strengthen arguments for allocating funds to rabies control policies. These must go together with awareness-raising campaigns for the different actors involved in rabies surveillance, so that animal owners and bite victims know when to suspect rabies and how to prevent it, and health professionals know how to proceed to confirm rabies suspected cases.

## Supporting information

S1 Data List of samples with test results. (XLSX)

S2 Data List of the10 samples collected in the field in Moramanga with test results. (XLSX)

S1 Fig Standards for reporting diagnostic accuracy flow diagram for RT-hn-PCR-T. (PDF)

S2 Fig Standards for reporting diagnostic accuracy flow diagram for RT-hn-PCR-FP. (PDF)

S3 Fig Phylogenetic trees of discordant samples 2555–15 and 3723–18. (DOCX)

S1 Table Standards for Reporting Diagnostic Accuracy checklist. (DOCX)
