# Supplementary material for: Enabling animal rabies diagnostic in low-access areas: Sensitivity and specificity of a molecular diagnostic test from cerebral tissue dried on filter paper
Source: PLoS Negl Trop Dis. 2020 Mar 6;14(3):e0008116. doi: 10.1371/journal.pntd.0008116 (PMC7135319; doi:10.1371/journal.pntd.0008116)
Supplement: S1 Fig — (PDF) [file pntd.0008116.s003.pdf]

Index test: RT-hn-PCR-T

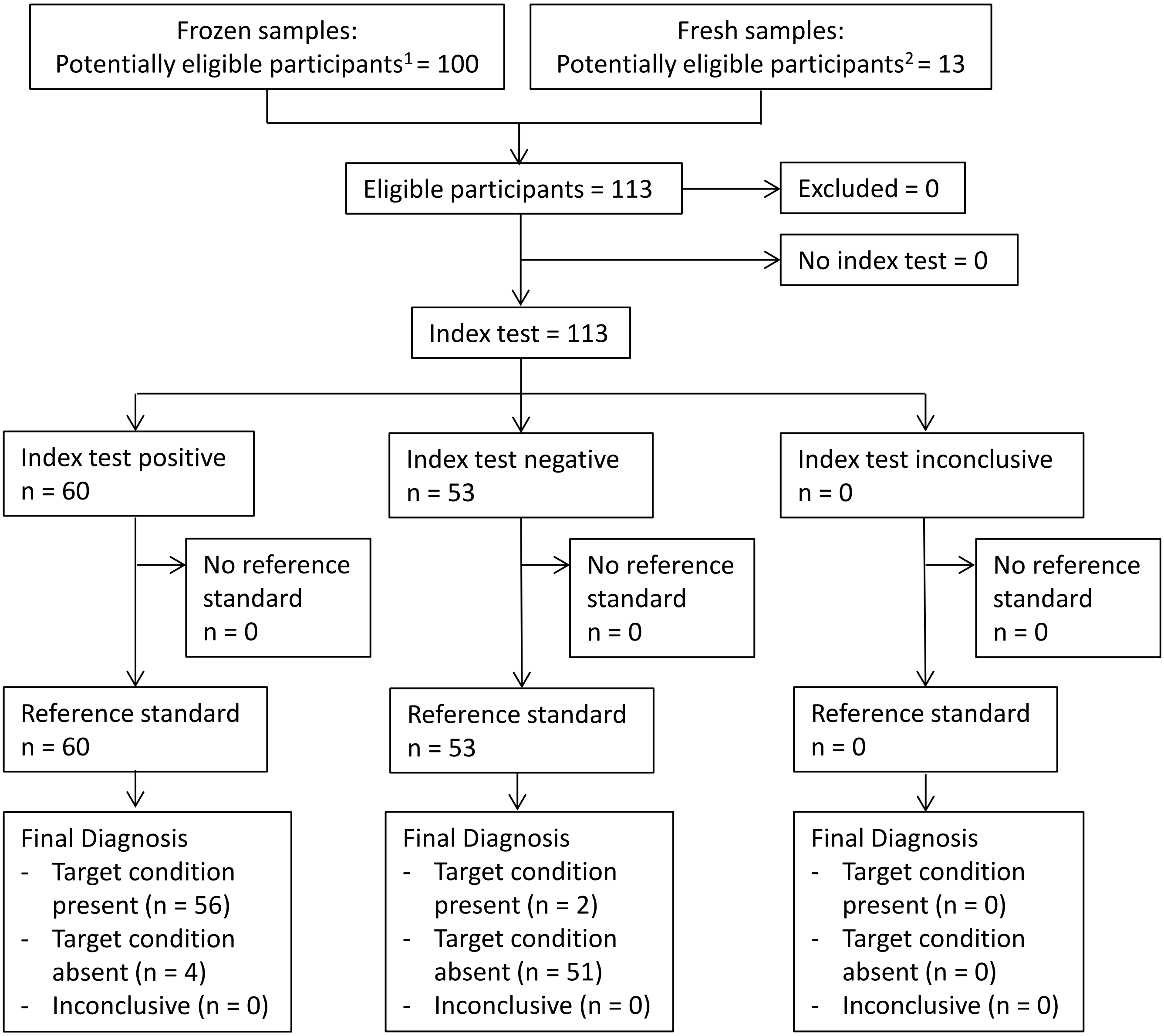

[1] *population: 100 most recent samples with sufficient volume, respecting approximatively a balance between FAT-positive and FAT-negative samples*

[2] *population: fresh samples arrived at the LNR during the study period*

S1 Fig: STARD (Standards for Reporting Diagnostic Accuracy) flow diagram for RT-hn-PCR-T
