# Supplementary material for: Enabling animal rabies diagnostic in low-access areas: Sensitivity and specificity of a molecular diagnostic test from cerebral tissue dried on filter paper
Source: PLoS Negl Trop Dis. 2020 Mar 6;14(3):e0008116. doi: 10.1371/journal.pntd.0008116 (PMC7135319; doi:10.1371/journal.pntd.0008116)
Supplement: S3 Fig — (DOCX) [file pntd.0008116.s005.docx]

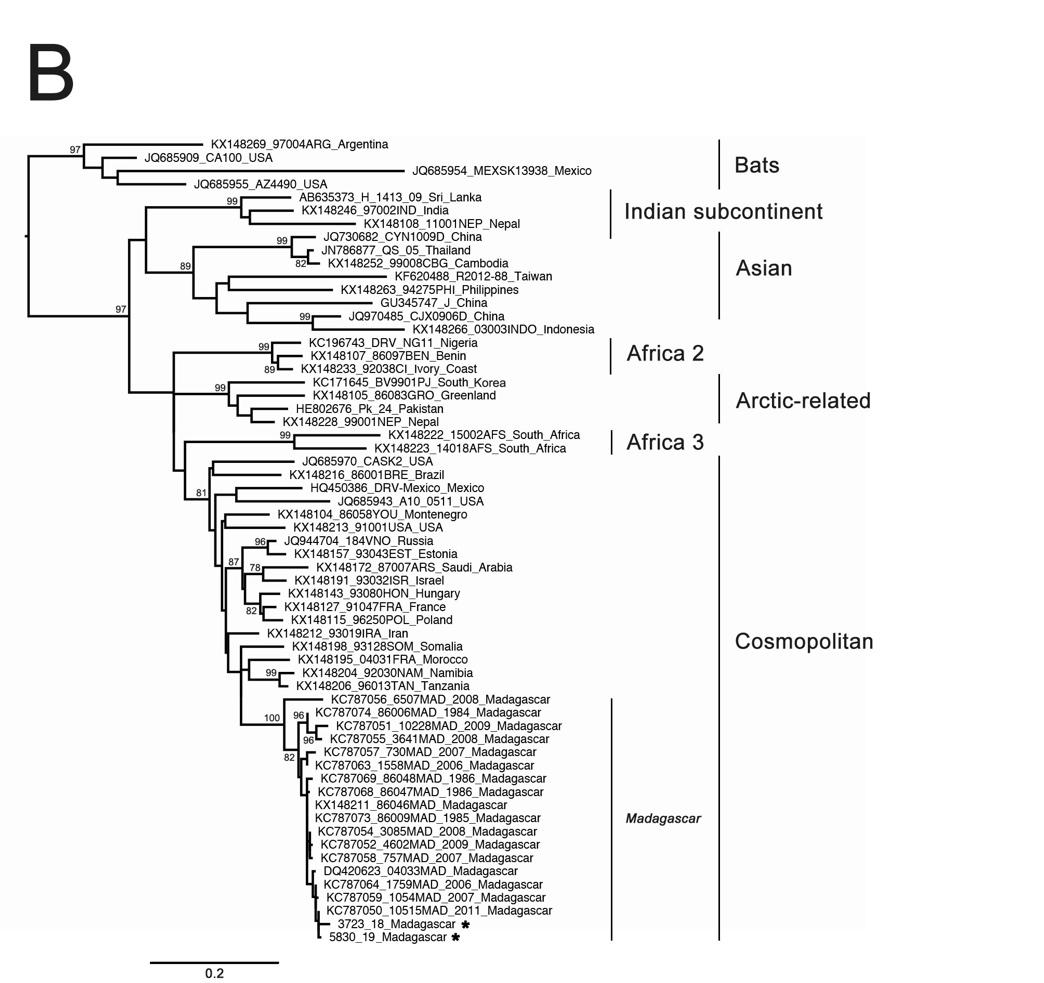

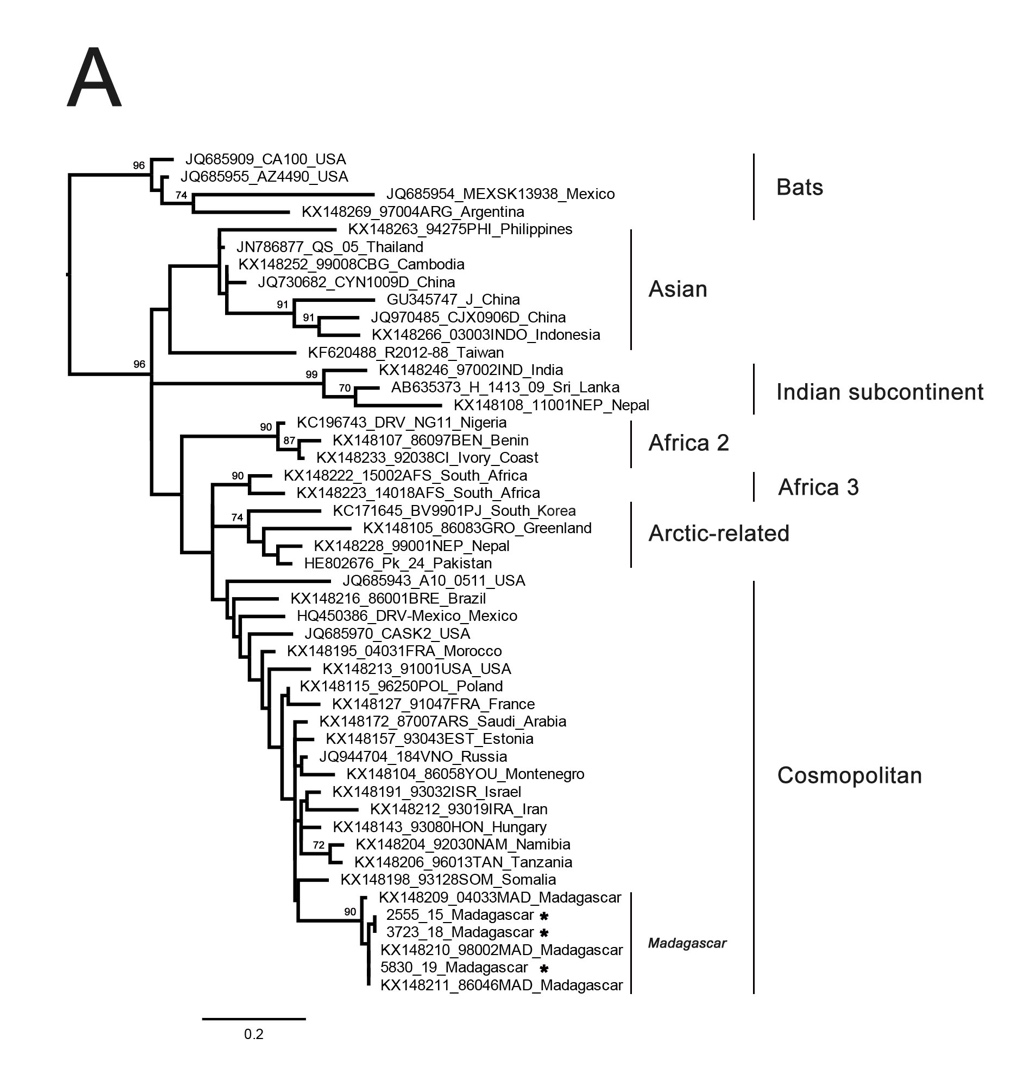
­

**S3 Fig: Phylogenetic trees of discordant samples 2555-15 and 3723-18.** Maximum-likelihood phylogenetic tree based on (**A**) partial polymerase (L) genes (208 nt) and on (**B**) partial nucleoprotein (N) genes (500 nt) of the discordant samples (2555-15 and 3723-18). Both of these samples were secondarily confirmed as positive after RT-qPCR and/or RT-hn-PCR, then Sanger sequencing at the National Reference Centre for Rabies (NRC-R) at Institut Pasteur, Paris, France. A representative dataset of sequences of rabies viruses (RABV) belonging to nearly all of the different existing clades previously defined [29] was included, as well as a subset of RABV sequences from Madagascar. In addition, the sequence of a positive Malagasy sample (5830-19) from the National Reference Laboratory (NRL) in Madagascar was also part of this subset of sequences. L-based (**A**) and N-based (**B)** trees were constructed using the maximum‐likelihood approach based on the TN93 model or based on the generalized time‐reversible model, respectively. Subtree pruning and regrafting (SPR) branch‐swapping and proportion of invariable sites plus gamma‐distributed rate heterogeneity (GTR+I+Γ4) were also selected for both models, after estimation by Smart Model Selection (SMS) with the Bayesian Information Criterion (BIC) substitution model in PhyML [31]. The robustness of individual nodes was estimated using 1000 bootstrap replicates with a Bayesian‐like transformation of aLRT (aBayes) [30]. Only bootstrap values ≥ 70 are indicated. Scale bar indicates nucleotide substitutions per site. Sequences generated in this study are indicated with an asterisk, and the main phylogenetic clades are mentioned. Sequence data are available from GenBank under accession numbers MN991293-MN991297.
