# Supplementary material for: Enabling animal rabies diagnostic in low-access areas: Sensitivity and specificity of a molecular diagnostic test from cerebral tissue dried on filter paper
Source: PLoS Negl Trop Dis. 2020 Mar 6;14(3):e0008116. doi: 10.1371/journal.pntd.0008116 (PMC7135319; doi:10.1371/journal.pntd.0008116)
Supplement: S1 Table — (DOCX) [file pntd.0008116.s006.docx]

S1 Table: STARD (Standards for Reporting Diagnostic Accuracy) checklist

| **Section & Topic** | **No** | **Item** | **Reported on page #** |
| --- | --- | --- | --- |
|  |  |  |  |
| **TITLE OR ABSTRACT** |  |  |  |
|  | **1** | Identification as a study of diagnostic accuracy using at least one measure of accuracy  (such as sensitivity, specificity, predictive values, or AUC) | Title |
| **ABSTRACT** |  |  |  |
|  | **2** | Structured summary of study design, methods, results, and conclusions  (for specific guidance, see STARD for Abstracts) | Abstract |
| **INTRODUCTION** |  |  |  |
|  | **3** | Scientific and clinical background, including the intended use and clinical role of the index test | Introduction, paragraphs 2-5 |
|  | **4** | Study objectives and hypotheses | Introduction, paragraph 5 |
| **METHODS** |  |  |  |
| *Study design* | **5** | Whether data collection was planned before the index test and reference standard  were performed (prospective study) or after (retrospective study) | Materials and Methods (M&M), paragraphs 1 & 2 |
| *Participants* | **6** | Eligibility criteria | M&M, paragraph 1 & 2 |
|  | **7** | On what basis potentially eligible participants were identified  (such as symptoms, results from previous tests, inclusion in registry) | M&M, paragraph 1 & 2 |
|  | **8** | Where and when potentially eligible participants were identified (setting, location and dates) | M&M, paragraph 1 & 2 |
|  | **9** | Whether participants formed a consecutive, random or convenience series | M&M, paragraph 1 & 2 |
| *Test methods* | **10a** | Index test, in sufficient detail to allow replication | M&M, paragraphs 2-11 |
|  | **10b** | Reference standard, in sufficient detail to allow replication | M&M, paragraph 2 & 8 |
|  | **11** | Rationale for choosing the reference standard (if alternatives exist) | Introduction, paragraph 5 |
|  | **12a** | Definition of and rationale for test positivity cut-offs or result categories  of the index test, distinguishing pre-specified from exploratory | M&M, paragraph 7-9 |
|  | **12b** | Definition of and rationale for test positivity cut-offs or result categories  of the reference standard, distinguishing pre-specified from exploratory | M&M, paragraph 8 |
|  | **13a** | Whether clinical information and reference standard results were available  to the performers/readers of the index test | M&M, paragraph 1 & 2 |
|  | **13b** | Whether clinical information and index test results were available  to the assessors of the reference standard | M&M, paragraph 1 & 2 |
| *Analysis* | **14** | Methods for estimating or comparing measures of diagnostic accuracy | M&M, paragraph 8 & 9 |
|  | **15** | How indeterminate index test or reference standard results were handled | M&M, paragraph 8 & 9 |
|  | **16** | How missing data on the index test and reference standard were handled | M&M, paragraph 2 |
|  | **17** | Any analyses of variability in diagnostic accuracy, distinguishing pre-specified from exploratory | M&M, paragraph 8 & 9 |
|  | **18** | Intended sample size and how it was determined | M&M, paragraph 1 |
| **RESULTS** |  |  |  |
| *Participants* | **19** | Flow of participants, using a diagram | Fig 1, S1 Figure and S2 Figure |
|  | **20** | Baseline demographic and clinical characteristics of participants | M&M, paragraph 1 |
|  | **21a** | Distribution of severity of disease in those with the target condition | NA |
|  | **21b** | Distribution of alternative diagnoses in those without the target condition | NA |
|  | **22** | Time interval and any clinical interventions between index test and reference standard | Fig 1 |
| *Test results* | **23** | Cross tabulation of the index test results (or their distribution)  by the results of the reference standard | Tables 1 and 2 |
|  | **24** | Estimates of diagnostic accuracy and their precision (such as 95% confidence intervals) | Results, Table 3 & paragraph 1 |
|  | **25** | Any adverse events from performing the index test or the reference standard | NA |
| **DISCUSSION** |  |  |  |
|  | **26** | Study limitations, including sources of potential bias, statistical uncertainty, and generalisability | Discussion, paragraphs 1-12 |
|  | **27** | Implications for practice, including the intended use and clinical role of the index test | Discussion, paragraphs 6-12, 15 |
| **OTHER INFORMATION** |  |  |  |
|  | **28** | Registration number and name of registry | NA |
|  | **29** | Where the full study protocol can be accessed | M&M |
|  | **30** | Sources of funding and other support; role of funders | Financial Disclosure |
|  |  |  |  |
